# 

**Published:** 1854-07

**Authors:** 


### INDEX.

PAGE.

A.

Americanized, another European principle, . . . . . 30

American Society of Dental Surgeons, meeting of, &c . 59

Auroplastic principle, artificial teeth, &c., . . . . . . 79

Application of teeth, and natural roots, &c., . . . . . 132

Artificial teeth, preparing the mouth for full sets, . . . . . 134

Action of gastric juice, . 145

Anatomy, a text book of, &c., . 163

American Journal of Science and Arts, . . . . . . 164

Auroplastic base, . . . 176

A new method for base, . . 178

Artificial teeth, by the auroplastic principle, . . . . . 223

Anatomy for the dentists, . 244

Alcoholic liquors, use and abuse of, &c., . . . . . . 322

Association of dentists, Mississippi Valley, . . . . . . 352

Address by Elisha Townsend, 374

Artificial teeth, new method of supporting, . . . . . 481

Artificial teeth, new method, . 442

Alcohol and its effects, . . 494

American Medical Monthly, . 301

Artificial teeth, improved sets of, 573

Artificial teeth, instruction in the use of, . . . . . 598

Arteries, elasticity of, . . . 664

B.

Bate, C. S., Esq., filling teeth, exposed pulp, . . . . 20
Bridgeman, M. K., osseous union, 33
Brown, Chas., compound fracture of lower jaw, . . . 34

PAGE.

Blandy, A. A., D. D. S., M. D. physical condition of the teeth, 38, 425
Bate, Spence, Esq., irregular teeth, &c., . . . . 93, 278, 282
Blow-pipe, . . . . . 132
Bile, . . . . . . . 149
Biliary secretions, . . . 149
Bull on the maternal management of children, . . . . 161
Base, a new method, . . . 178
Blow-pipe, hot air, . . . 238
Buckingham, S. L., M. D., . 265
Blood, temperature of, . . 317
Bronchitis and pneumonia, . 323
Badger, F. H., Dr., . . . 331
Bellows, blow-pipe and forge, . 658
Brandy and red noses, . 674

C.

Chemistry of the metals, 3, 181, 337
Case of compound fracture of the lower jaw, . . . . . 34
Condition of the teeth, physical, &c., . . . . . . . 38
Caries of the teeth, . . . . 129
Chemical characters of gastric juice, 145
Chloroform, . . . . . 154
Children, management of, . . 161
Chemistry, metallurgy, &c., . 166
Chloroform inhalations, . . 178
Corundum files, &c. . . 180
Chemistry for dentists, . . 232
Chemistry, organic and physiological, . . . . . . . 254
Chloroform, death from, . 272, 275
Carious teeth, diseases from, . 278
Caustic, a new, . . . . 289
Caries of the teeth, . . . 308
Clasps, . . . . . . . 310
Cell growth, . . . . . 314
Capillaries of the liver, . . 321

PAGE.
Cartilage, dentine bone, structure of, . . . . 321
Chemistry, elements of, for, . 325
Chemistry, handbook of, . 329
Chemistry, principles of . . 331
Cholera, . . . . 333
Clerical gullibility, . . . 335
Chloroform, concerning, . 565
Caries and exfoliation of jaw, . 567
Cellulose in the human body, 662
Chemical lectures on consumption, 667
Circulation, theory of, . . 671
Commencement of the Baltimore College of Dental Surgeons, 675

D.

Dental medicine, memoirs of a few fundamental points, &c., . 105
Dental pulps, treatment of, &c., 122
Dentine, on inflammation of, 124
Dentists, slandering each other, 172
Dental Expositor, . . 179
Dental practice, on . 259
Dentistry, mechanical, . . 265
Development of the teeth, . 291
Dental medicine, . 294, 637
Dental patents, . . 302
Deposition of ivory, in the, &c., 307
Development of blood globules, 316
Dental pulp, treatment of, . 305
Dentistry, practice of, . . 312
Diamond, artificial, . . 334
Dwinelle, W. H , M. D., D. D. S., 346
Dental pulp, dissertation on, by Chapin A. Harris, . . 388
Dental tissue, essay on, by T. Russel, . . . . 405
Dentistry in France, . . 486
Dentistry at Kaw Mendi, West Africa, . . . . 490
Dental education, . 501, 628
Dental Recorder, . . 503
Dental Surgeons, M. V. Ass'n of, 552
Dubs, C. H., . . 571
Dental science, . . . 647
Decay of teeth, periods of, . 655

E.

Extraction of wisdom tooth, tumor, 35
Etiquette, professional, . 51
Extraction of teeth, . . 124
Essay on the mortality of children, 243
Evans, T. W., D. D. S., 259, 448
Electricity, relations of, . 320
Exostosis. By Harvey, . 556

PAGE.
F.

Filling teeth when complicated with inflammation of the pulp, 20
Fracture of lower jaw, . 34
Filling teeth over exposed nerves, 124
Fusil oil, . . . 156
Freak of nature, . . 307
Fitzpatrick, Dr. A. . . 442
French, M. D., D. D. S., on, &c., 531
Flagg, Josiah Foster, biographical notice of, . . . 592
Freak of nature, . . 649
Fat in sugar, relations of, . 665
Flagg, J. F., . . 674

G.

Gastric secretions, . . 143
Gastric juice, . . . 145
Gastric juice, action of, . 147
Gastric juice, quantity of, . 148
Gold and silver, on parting, 227
Gold, crystallized, . 333, 653
Gold sponge, . . 346, 425
Georgia Blister and Critic, . 501
Glottis, movements of, . 662

H.

Hunter, W. M., European principle Americanized, . . 30
Harris, Chapin A., spontaneous inflammation of the dental pulp, 48
Harris, Chapin A., porcelain teeth mounted, &c., . . 55
Hill, A., on auroplastic principle, 223
Handy, W. R., M. D., . . 244
Hair lip, . . . . 286
Histolysis . . . 315
Heart, function of, . . 318
Harris, C A., M. D., D. D. S., on dental pulp, . . 378, 516
Huxley, T. H., . . . 462
Hearing, restoration of, . 490
Heart, movements of, . . 498
Homœopathy, its tenets, &c., 499, 570
Homœopathy, fairly represented, 500
Harrison, J. D., . . 565
Harvey, Dr. S. H., . 566

I.

Inflammation of the pulp, . 20
Inflammation, spontaneous, of the alveolo-dental membrane, 48
Irregular teeth, . . . 93
Inflammation of dentine, . 124
Irregularity of the teeth, . 129
Intermittent odontalgia, . 132

PAGE.
Intestinal juice, . . . 153
Iowa Medical Journal, . 168
Ice, as a local anæsthetic, . 180
Inflammation of the lips and face, 623

J.

Johnson, Christopher, M. D., upon the teeth, &c., . 63, 238
Juice, gastric, . 145, 147, 148
Juice, pancreatic, . . 151
Juice, intestinal, . . 153
Journal of Science and Arts, 328, 669

K.

Keys, J. W., patents, &c., . 14
Koelliker, Dr. A., microscopic anatomy, &c., . . . 63

L.

Loss of teeth, &c., . . 164
Lost at sea, . . . 179
Lewis, John, D. D. S., mechanical dentistry, . . . 191
Levison, J. L., D. D. S., on tobacco, &c., . . 240, 248, 562
Lectures on surgical pathology, 323
Lecture on venereal diseases, 324
Lecture, introductory, Yale college, 330
Leslie, M., D. D. S., F. M. V. A. 352
Loomis, M., . . 573
Lusus naturæ, . . . 658
Lime, phosphate of, . 665

M.

Meeting of American Society of Dental Surgeons, . . 59

Microscopic anatomy on histology of man, . . . 63

Medicine, dental, . . 105

Microscopist the, or a complete manual on the use of the microscope, . . . 162

Maxillary bone, reproduction of, 171

Mechanical dentistry, . . 191

Mortality of children, . 243

Molar, lower, with four roots, . 333

Mustaches, . . . 334

Mechanical dentistry, by M. D. French, . . . 531

Maxillary bone, removal of, 652

N.

Nicotin, . . . 159
Nitrate of silver, . . 657
News Letter, . . . 672

PAGE.

O.

Odontalgia, intermittent, . 132
Odontalgic remedy, . . 134
Oil, fusel, . . . 156
Opthalmic surgery, treatise on, 165
Observations on the disease and loss of teeth, . . . 167
Our new volume, . . 168
Obituary, . . 504, 676
Ossification of the pulp, . 673

P.

Patents and patentees, . . 14
Principle Americanized, . 30
Physical condition of the teeth, &c., 38
Professional etiquette, . 51
Porcelain teeth mounted, &c., . 55
Plates, springing of, . 131
Plate teeth, . . . 131
Plates, swaging, . . 134
Preparation of mouth for full sets, 134
Pancreatic juice, . . 151
Poisons, organic, . . 156
Portrait of Professor White, 179
Piggot, A. S., M. D., on gold and silver, . . . 227
Palate, hard, destruction of, . 282
Practice of dentistry . 312
Platina pivot, . . . 307
Pain in the teeth and jaws, . 313
Phrenic nerve, . . . 318
Pneumogastric nerve, . 319
Periostitis, . . . 492
Periosteal irritation, case of, 493
Pancreatic fluid, . . 498
Patron to the dentists, . 504
Presence of mind, advantages of, 562
Pulse, influence of respiration, 660
Prize, . . . 670

R.

Risodontryphy, . . 134, 489
Reproduction in the maxillary bone, 171
Russell, W. S., D. D. S., M. D., 405
Rock, J. S., M. D., . . 628

S.

Silver and compounds, . 3
Scott, F. C., tumor cured, &c, 35
Spontaneous inflammation of the alveolo-dental membrane, . 48
Springing of plates, . 131
Swaging plate, . . . 134
Saliva, . . . 137
Secretions, gastric, . . 143

Murray

Page.
Secretions, biliary, . . . 149
Summary, . . . 173
Sponge gold, 174, 346, 425, 650
Spotted child, . . . 180
Salivary, on elective elimination of, &c., . . . 290
Supernumerary teeth, . . 313
Stokes, C., M. R. C. S., . 418
Spiritualism, . . . 429
Sugar grape, . . 497
Short comings, . . . 504
Shelby, Dr. W. A., . . 567
Suction plates, improvement in, 571
Spiral springs, a new apparatus for, 574
Schmedicke, C. N. L., . 574
Soda water, poisonous effects of, 642
Soldering teeth, . . 655
Surgery, science and art of, . 668

T.

Teeth, union of, . . 33
Tumor of two years standing, cured by extraction of wisdom teeth, 34
Teeth, physical condition of, &c., 38
Teeth, porcelain, mounted, &c. 55
Truman, Edwin, on auroplastic principle, . . . 79
Teeth, on irregular, . 93
Talma, A. F., M. D., on dental medicine, . . . 105
Treatment of exposed pulp, 122
Teeth, filling on exposed nerves, 124
Teeth, extraction of, . . 124
Teeth, irregularity of, . 129
Teeth plate, . . . 129
Teeth, caries of, . . 131
Teeth, on plate, to roots of natural teeth, . . . 132
Teeth, in respect to character, . 135
Tobacco, . . . 157
Teeth, on the loss of, &c., . 164
Third Editor, . . 170
Tax on lawyers, dentists, &c., . 180
Tobacco, oil of, . . . 240
Tooth drawing, . . 311
Thackston, W. W. H., M. D., D. D. S., . . . . 362
Townsend, E., D. D. S., M. D., 374
Teeth, cleansing the, by Thompson, 423
Thompson, T. D. . . 423
Teeth, regulation of, . . 448
Teeth, development of, . . 462
Teeth, treatment of, . 488
Tomes, John, F. R. S., . 598
Tongue, black fur on the, . 633
Tooth-ache, . . . 659
Teeth, repair of, . . 664
Turkey and the Turks, . . 669
Townsend, resignation of, . 674
Trouble in the wigwam, . 676

U.

Union of teeth, . . . 33

V.

Venereal diseases, . . 324
Vesico-vaginal fistula, . . 327
Valedictory, by Thackston, . 362

W.

Wright, Prof. R. N., chemistry of metals, &c., . 3, 181, 337, 505

Y.

Yellow fever, . . . 330

